# First Initial community-acquired meningitis due to extended-spectrum beta-lactamase producing *Escherichia coli *complicated with multiple aortic mycotic aneurysms

**DOI:** 10.1186/1476-0711-11-4

**Published:** 2012-02-09

**Authors:** Pierre Weyrich, Nicolas Ettahar, Laurence Legout, Agnes Meybeck, Olivier Leroy, Eric Senneville

**Affiliations:** 1 Infectious diseases department, Dron hospital, Tourcoing, France; 2 Intensive care unit, Dron Hospital Tourcoing, France

**Keywords:** aortic mycotic aneurysm, ESBL producing *Escherichia coli*, meningitis

## Abstract

We report the first case of extended-spectrum beta-lactamase producing *E. coli *community-acquired meningitis complicated with multiple aortic mycotic aneurysms. Because of the acute aneurysm expansion with possible impending rupture on 2 abdominal CT scan, the patient underwent prompt vascular surgery and broad spectrum antibiotic therapy but he died of a hemorrhagic shock. Extended-spectrum beta-lactamase producing *E. coli *was identified from both blood and cerebrospinal fluid culture before vascular treatment. The present case report does not however change the guidelines of Gram negative bacteria meningitis in adults.

## Introduction

Adult community-acquired meningitis due to *E. coli *is a rare entity. It generally occurs in patients with compromised immune status or cirrhosis. When direct examination of the cerebrospinal fluid (CSF) shows the presence of Gram negative bacilli (GNB), current guidelines recommend the use of a 3^rd ^generation cephalosporin (cefotaxime or ceftriaxone) [1,2]. We herein report the first case of extended-spectrum beta-lactamase (ESBL) producing *E. coli *community-acquired meningitis complicated with multiple aortic mycotic aneurysms.

## Observation

In September 2010, a 59-year-old patient with a history of chronic alcohol and tobacco consumption was admitted to the emergency unit for consciousness disorders and fever. Two days before his admission, the patient had presented headache and nausea.

**Figure 1:**
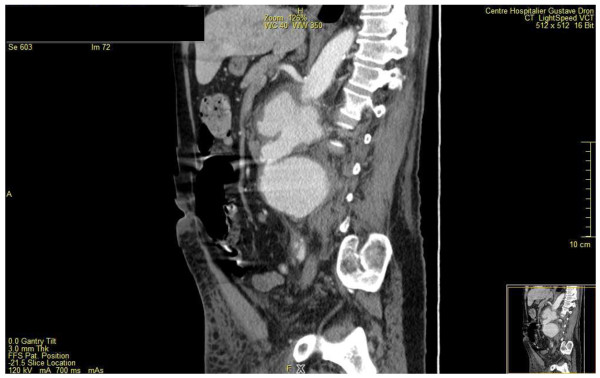
**multiple mycotic aneurysms of the infra-renal aorta**.

**Figure 2:**
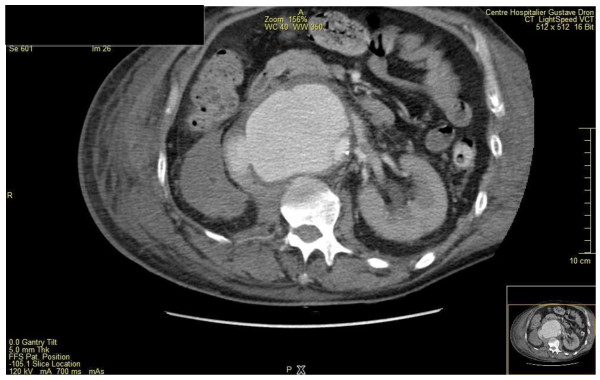
**multiple mycotic aneurysms of the infra-renal aorta and inferior vena cava thrombosis, incomplete right kidney infarct and a delay in left kidney perfusion**.

At admission (09/28), physical examination revealed a frank meningeal irritation, consciousness disorders with a Glasgow Coma Scale (GCS) of 12. The patient's hemodynamic status was stable and, no other physical abnormality was found. WBC count was 3.85 G/L; hemoglobin rate, 14.6 g/dl; platelets count, 64 G/L; C-reactive protein rate (CRP), 292 mg/L; procalcitonin rate, 21.9 ng/ml and prothrombine rate, 44%. The renal and hepatic functions were normal. CSF examination showed 440 cells/mm3, (neutrophils 62%, lymphocytes 29%) with a glucose and protein rate at 0.01 mmol/l and 10.35 g/l, respectively. The chest X-ray, electrocardiogram and cerebral computer tomography (CT) scan were normal. The patient was transferred to the intensive care unit few hours after his admission because of a rapid deterioration of consciousness (GCS 6) and the occurrence of septic shock. Mechanical ventilation, volume resuscitation, hydrocortisone hemisuccinate, vasopressors, platelets transfusion and intravenous empirical broad spectrum antibiotic therapy were administered. The patient received cefotaxime 18 g/24 h, amoxicillin 12 g/24 h and gentamicin 460 mg/24 h. Both CSF and blood culture yielded an ESBL producing *E. coli *resistant to cefalotin (MIC ≥ 64), cefotaxime (MIC ≥ 64), ceftriaxone (MIC ≥ 64), cefixime (MIC2), intermediate to cefepim (MIC 2) and ceftazidime (MIC 2), and susceptible to cefoxitin (MIC ≤ 4), ertapenem (MIC ≤ 0.5), meropenem (MIC ≤ 0.5), imipenem (MIC ≤ 1), gentamicin (MIC ≤ 1), ofloxacin (MIC ≤ 0.12)and ciprofloxacin (MIC ≤ 0.25). Cefotaxime was switched to meropenem (6 g per day) combined with ciprofloxacin (1.2 g per day). No abscess was found on brain MRI and thoraco-abdominal CT scan. Meropenem-ciprofloxacin therapy was discontinued after 21 days of treatment (10/18). The patient's condition improved slowly, allowing his extubation after 20 days of mechanical ventilation (10/18). Abnormal CRP rate (> 120 mg/l) persisted at this moment. One week after extubation (10/25), the patient was transferred to the department of infectious diseases. At the admission, physical exam showed confusion with slow ideation. There was no abnormality on cardiac, pulmonary and urological examination except a moderate abdominal painful at palpation. Ten days after stopping the antimicrobial treatment (10/28), the laboratory tests showed a persisting elevated rate of CRP (212 mg/L) and a elevated white blood cell count to 23.6 G/L. Thoracic and abdomi-nopelvic CT scan were performed which showed multiple mycotic aneurysms of the infra-renal aorta, the aorto-iliac bifurcation and the primitive iliac artery, an inferior vena cava thrombosis, a complete right kidney infarct and a delay in left kidney perfusion. (Figure 1 and 2). The transthoracic echocardiography showed no sign of infectious endocarditis. The patient was transfered to the operating room for aorto-iliac by-pass. No bacteria growth on new bacterial samples but there was taken after antibiotic treatment was started. Unfortunately, the patient died during the surgery of a hemorrhagic shock.

## Discussion

Community-acquired meningitis in adults due to *E. coli *is a rare entity. The main risk factors are alcoholism, cirrhosis, neoplasic diseases, diabetes mellitus, and treatment with immunosuppressive agents [3-7]. Others cases occur frequently in neurosurgery and are usually associated with multi-drug resistant strains [8-11]. In our observation, the patient had no risk factors except a chronic alcoholism and perhaps his dog as animal pet.

In Europe, there is an increasing in 3^rd ^generation cephalosporin resistant *E. coli *isolates in both hospital setting and the community. CTX-M ESBL is the most common genetic support of resistance in these strains [12]. In our case report, we suspected this mechanism. The spread of *E. coli *producing ESBL is now well- identified [13].

Blood stream infections due to *E. coli *in adults are often related to underlying urinary or bilary tract or other intra-abdominal infections. Vascular infections and meningitis due to *E. coli *are exceedingly rare and few cases isolated have been reported [14-16]. Diabetes mellitus, previous fluoroquinolone use, recurrent urinary tract infection, previous hospital admission and older age in male patients have been identified as risk factors for infection of ESBL-producing *E. coli *[17]. Recently, Ewers and al. suggested the possibility of inter-species transmission of multiresistant strains of *E. coli *from human to animal and vice versa [18].

The current guidelines for the management of gram negative bacilli (GNB) related meningitis are well codified and recommend the use of a 3^rd ^generation cephalosporins ± gentamicin. Alternative therapies are Cefepime, meropenem, aztreonam, fluoroquinolone, and trimethoprim-sulfamethoxazole [1,2]. In our case report, meropenem (in association with ciprofloxacin) was used because of the lower risk of seizures compared to imipenem [19,20]. Mortality associated with GNB related meningitis varied from 25 to 100% [7,21]. Lu et al. identified several risk factors for mortality associated to GNB related meningitis: inappropriate initial treatment, septic shock, initial level of consciousness, hyperosmolar hyperglycemic coma, disseminated intravascular coagulation, high CSF lactate levels and leucocytosis. In the multiple logistic regression analysis, only appropriate antimicrobial therapy and septic shock were strongly associated with mortality even after adjusting for other potentially confounding factors [21]. Inappropriate treatment and septic shock were initially present in our patient. The presence of a concomitant aortic mycotic aneurysm was an additional factor of mortality.

Only two cases of mycotic aneurysm in patient presenting initially bacterial meningitis have been reported [22,23]. The first case is a 65-year old woman with ruptured mycotic aneurysm in a patient with pneumococcal meningitis, who died on week later. The second case is a 57 year-old man with a history of CFS fistula and multiple neurosurgical treatment, who developed meningitis complicated with endocarditis and thoracic aortic infection. No bacteria were identified in this second case. The patient was successfully treated with endovascular prosthesis. In the present case report, multiple mycotic aneurysms were suspected because of the sustained bacteremia, the atypical and multiple foci of vascular infection, the normality of the first abdominal CT scan and the rapid evolution of aneurysm. the aortic involvement may be the result of infection of the aortic wall.

## Conclusion

The present case report highlights the risk of meningitis due to ESBL producing *E. coli *in a patient with apparently no risk factor for this infection with this type of resistant bacilli. This unique observation does not provide, however to modify the current guidelines for the treatment of community acquired meningitis in adults.

## Consent

Written informed consent was obtained from the patient's family for publication of this case report and accompanying images.

## Competing interests

LL received speaking honoraria from Novartis.ES received speaking honoraria from Pfizer and Novartis. OL received speaking honoraria from Pfizer and Novartis. PW, NE and AM had no conflict of interest.

## Authors' contributions

PW, LL, OL, ES participated in the design of the study. LL conceived the case report and participated in its design and coordination. NE and AM collected the data. All authors read and approved the final manuscript.
